# miRTissue: a web application for the analysis of miRNA-target interactions in human tissues

**DOI:** 10.1186/s12859-018-2418-5

**Published:** 2018-11-30

**Authors:** Antonino Fiannaca, Massimo La Rosa, Laura La Paglia, Alfonso Urso

**Affiliations:** CNR-ICAR, National Research Council of Italy, Via Ugo La Malfa, 153, Palermo, 90146 Italy

**Keywords:** miRNA-target interaction, Protein expression value, Human tissue, TCGA, Cancer type

## Abstract

**Background:**

microRNAs act as regulators of gene expression interacting with their gene targets. Current bioinformatics services, such as databases of validated miRNA-target interactions and prediction tools, usually provide interactions without any information about what tissue that interaction is more likely to appear nor information about the type of interactions, causing mRNA degradation or translation inhibition respectively.

**Results:**

In this work, we introduce miRTissue, a web application that combines validated miRNA-target interactions with statistical correlation among expression profiles of miRNAs, genes and proteins in 15 different human tissues. Validated interactions are taken from the miRTarBase database, while expression profiles are downloaded from The Cancer Genome Atlas repository. As a result, the service provides a tissue-specific characterisation of each couple of miRNA and gene together with its statistical significance (*p*-value). The inclusion of protein data also allows providing the type of interaction. Moreover, miRTissue offers several views for analysing interactions, focusing for example on the comparison between different cancer types or different tissue conditions. All the results are freely downloadable in the most common formats.

**Conclusions:**

miRTissue fills a gap concerning current bioinformatics services related to miRNA-target interactions because it provides a tissue-specific context to each validated interaction and the type of interaction itself. miRTissue is easily browsable allowing the user to select miRNAs, genes, cancer types and tissue conditions. The results can be sorted according to p-values to immediately identify those interactions that are more likely to occur in a given tissue. miRTissue is available at http://tblab.pa.icar.cnr.it/mirtissue.html.

**Electronic supplementary material:**

The online version of this article (10.1186/s12859-018-2418-5) contains supplementary material, which is available to authorized users.

## Background

One of the main focuses of translational medicine is the comprehension of molecular mechanisms that characterise the cellular behaviour of complex human diseases [1]. microRNAs (miRNAs) are “master regulators” of gene expression [2], and several pieces of evidence show their involvement in physiological and pathological processes by interaction with target genes [3, 4]. In cancer, one of the main relevant aspects of miRNAs is that they can act as “oncogenes” or “tumor suppressor” genes depending on which target they bind and the cellular environment [5–8]. Moreover, there are several in vitro functional studies evidencing this dual role of miRNAs in tumorigenesis. As an example, the over-expression of mir-17-92 cluster, considered as an oncogene, is related to lymphoproliferative malignancies. The over-expression of let-7 tumor-suppressor is, in turn, related to reduced tumor burden [9]. Also in breast cancer miRNAs are considered promising molecular “biomarkers” as their profiling can be associated with different breast cancer subtypes, helping to differentiate patients by the different response to therapies, and improving this way the clinical management of patients [10, 11].

**Fig. 1:**
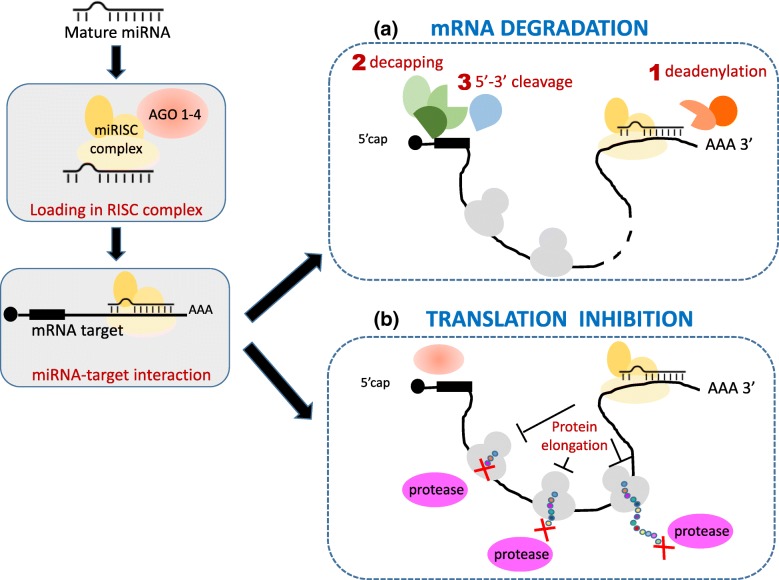
Mechanism of miRNA-target interaction. **a** mRNA degradation and **b** translation inhibition schema. **a** mRNA degradation: after binding miRISC complex, there is a recruitment of a deadenylase complex (CAF1-CCR4-NOT) acting on 3’UTR region of mRNA. Poli A tail is then removed [47, 48]. After deadenylation, decapping of 5’UTR may occur through a synergistic action of different protein factors (DCP1, DCP2, DDX6, EDC4) [47]. Finally 5’-3’ exonucleases lead mRNA degradation [49]. **b** Translation inhibition: Translation repression is due to miRNA intervention in different steps of translation. AGO protein has been showed to compete with 5’capping protein factors [50], blocking translation at initiation step. Other mechanisms of miRNA action involve elongation step, causing a premature protein termination [51]

**Fig. 2:**
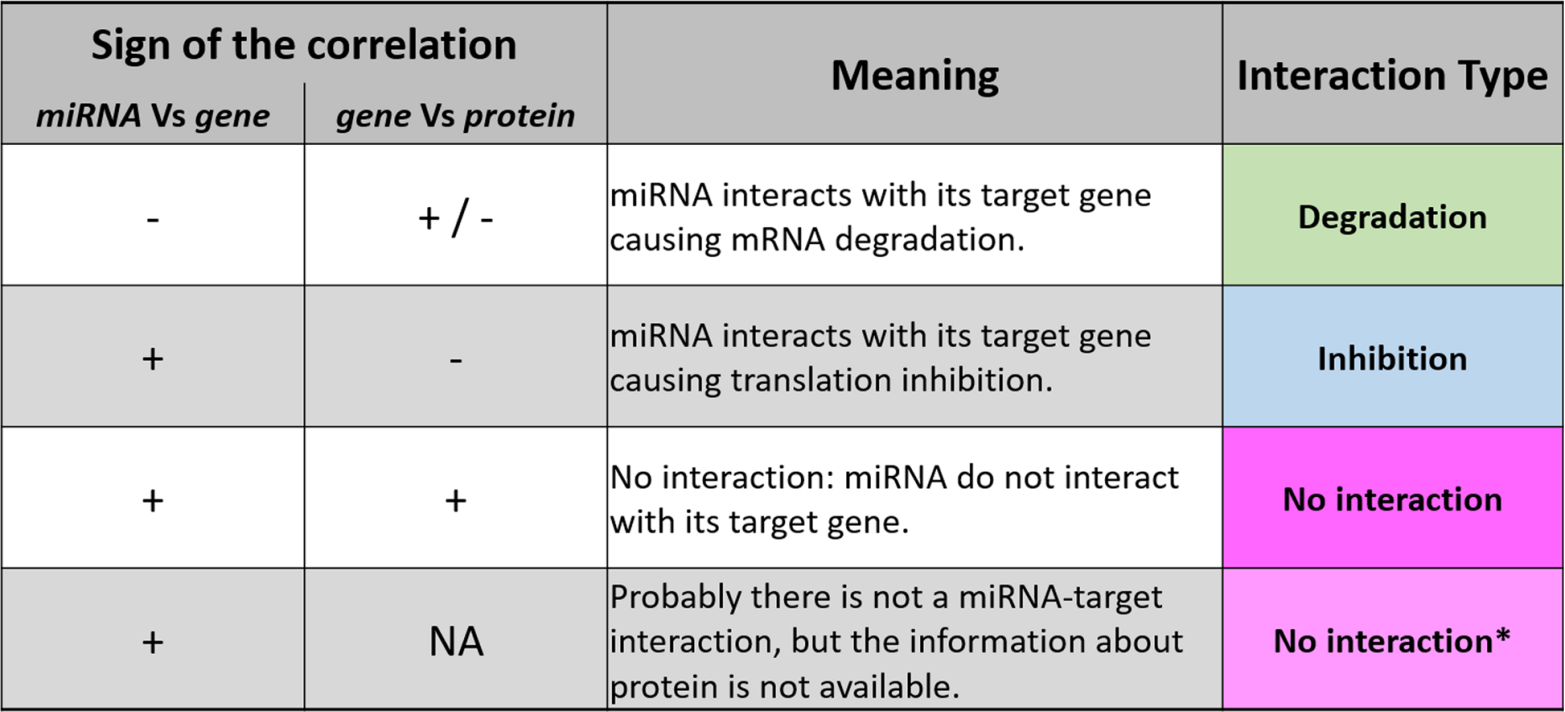
miRNA-target interaction types. According to the biological meaning of the correlation signs among miRNA, gene and protein expression values, we report four types of miRNA and gene interactions

miRNAs act on target gene through the interaction with a target sequence within RNA messenger (mRNA). The interaction occurs mainly through the recognition and imperfect binding of 3’ untranslated region (UTR) of mRNA, and also (less frequently reported) of the 5’ UTR and coding sequences (CDS) regions [12–14]. The main portion of miRNA sequence interacting with mRNA target is called “seed sequence”, and it is 6-8 nucleotide long [15]. However, the remaining part of the small non-coding RNA contributes to making miRNA-target bond stable. Depending on the bond strength between two interacting molecules and consequently on the degree of homology with the target site, miRNA-target interaction can lead to two different mechanisms [16]: (1) miRNA can lead to mRNA cleavage, and consequently its degradation (Fig. 1a), or (2) miRNA can block protein translation process (Fig. 1b). The second mechanism is detectable by looking at the abundance levels of proteins, and not only of transcripts. In other words, the abundance of proteins in tissue can play a fundamental role in the miRNA-target analysis.

Bioinformatics services and tools, such as databases (db) of validated miRNA-target interactions as well as miRNA-target predictors [17, 18] provided a significant contribution to the investigation of miRNA-target interactions. In this topic, one of the main obstacles is to achieve a high grade of specificity and sensitivity [19]. To overcome this issue, some predictors have introduced experimentally validated interactions. One example is DIANA-MicroT-CDS predictor [20]. It provides miRNA-target interactions both predicted and validated by in vitro experiments, through the support of TarBase db [21]. The latter is a manually curated miRNA-target database, experimentally supported and it includes targets derived from high throughput experiments. Although validations improve the results of interactions, it still lacks information about tissue-specificity because no hint is given about which tissue is more likely to exhibit a predicted and/or validated interaction. MiRWalk system [22] also provides predicted and validated miRNA-target interactions, but it also lacks tissue information. Although the introduction of experimental approaches reduced the number of false positive predictions, that biological information is still quite incomplete, as not all tissues express the same molecules (miRNA and mRNA) at the same time. Moreover, there is the need to have more information on interactions between RNA molecules in a specific case study, and in one particular tissue context.

As an example, we report the case of a miRNA that is over-expressed in a specific tissue type or a certain cellular condition, but its predicted target is not expressed in the same tissue. The produced effect on the cell’s phenotype of such a miRNA can be expected to be rather small. Of course, to overcome this evidence, a validation approach is needed. In particular, the inclusion of expression values in such bioinformatics methods could help to define the molecular interactions between the discussed molecules better. Indeed, recently few miRNA-target prediction methods began to integrate the expression levels of both RNA molecules [23, 24]. For example, the ComiR algorithm [25] uses user-provided miRNA expression levels together with thermodynamic modelling and machine learning techniques to make more accurate predictions, but no straight miRNA-target couples can be evidenced and analysed. miRTarBase [24] is an experimentally validated miRNA-target interaction db, which provides tissue information about miRNA-target interactions, by considering Pearson correlation between miRNA and gene expression profiles. Another exciting predictor is miRGator [26]. It uses both validated interactions and expression values to characterise interactions with regards to specific tumor tissues, by means once again of Pearson correlation. All the above-described bioinformatics tools lack, however, data regarding protein abundance. That kind of data, in fact, is needed to predict interactions causing protein translation inhibition and that, therefore, can not be predicted only considering miRNA and mRNA expression profiles.

In this paper, we present miRTissue, a web application that can provide, for 15 types of human tissues (both tumor and normal), the type of miRNA-target interaction of those validated pairs. To do that, miRTissue exploits the statistical correlation among expression profiles of miRNAs, genes and proteins. In this way, it is possible to have lesser false positive, removing validated interaction not statistically related to a specific tissue, and at the same time to have more sensitive and specific responses. These features can provide a close view of the miRNA status of cells, tissues or organisms. Moreover, miRTissue is the first service that includes protein expression values for the analysis of miRNA-target interactions, in order to provide a novel insight into the type of interactions.

## Methods

In this section, we introduce databases, software libraries and algorithms we used to obtain additional information on miRNA-target interactions. Besides, we introduce the development environment we used to implement miRTissue, the proposed web application visualising the results. For further and in-deep implementation details please refer to Additional file 1.

### miRTarBase DB

miRTarBase is one of the complete databases collecting annotated and experimentally validated miRNA-target interactions [24]. Starting from a pre-screening obtained using text mining technique of research articles, validated miRNA-target interactions are extracted from reporter assay or western blots (strong validated interactions) and CLIP-seq datasets. In this work, we used miRTarBase vers. 7.0, comprising about 380000 validated miRNA-target interactions related to human species.

### TCGA

The cancer genome atlas (TCGA, https://cancergenome.nih.gov/) is a research program carried out by a collaboration between the National Human Genome Research Institute (NHGRI) and National Cancer Institute (NCI) with the aim of identifying changes in each cancer’s genome to understand the underlying mechanism that causes the disease. TCGA dataset stores about 2.5 petabytes of data obtained from tumor and normal tissues originating from more than 11000 patients; it can be considered the reference database for this kind of data. Collected data include, for example, expression profiles of miRNAs, genes, proteins belonging to both tumor tissues and normal ones. In this work, we downloaded miRNA, gene and protein expression values from the TCGA database. As regards miRNA and gene expression quantification data, we considered only RNA-Seq experimental strategy data for both “solid tissue normal” and “primary solid tumor” samples. Concerning protein expression data, since normal tissue protein expression values are not available in TGCA database, we took into account only “primary solid tumor” samples from the TCGA legacy archive. In any case, we considered FPKM normalisation for all expression data. The tissue types, normal and tumor, and their corresponding number of the sample with miRNA, gene and protein expression profiles are summarised in Table 1.

**Table 1:** Number of samples with miRNA, gene and protein expression values in TCGA database (*release 2016-05-15*)

PROJECT	NORMAL			TUMOR		
	miRNA	Gene	Protein	miRNA	Gene	Protein
ACC	0	0	0	80	79	46
BLCA	20	20	0	347	345	339
BRCA	35	35	35	872	870	860
CESC	3	3	0	307	304	173
CHOL	9	9	0	36	36	30
COAD	8	41	0	444	456	360
DLBC	0	0	0	47	48	33
HNSC	44	44	0	523	500	357
KICH	25	24	0	66	65	63
KIRK	71	72	0	516	530	478
KIRP	34	32	0	291	288	215
LIHC	50	50	0	368	370	177
LUAD	46	59	0	519	533	359
PRAD	52	52	0	495	495	352
UCEC	33	35	0	538	543	440

### Global test

The global test is a statistical test used to measure the correlation between one or more features, called covariates, and a response variable [27]. The global test is based on the null hypothesis that none of the covariates is correlated with the response variable. The alternative hypothesis is that at least one of the covariate is correlated with the response. To find that correlation, the global test uses a regression model that models the distribution of the response as a function of the covariates. The global test can also be used on a set of features, to test their association with a response variable. In its original formulation [27], the global test has been adopted to examine the association between a group of genes and a clinical outcome. In a more recent work [28], authors used the global test to measure the correlation between the expression profile of a miRNA and the expression profiles of the group of its target genes. The contribution of each target gene to the global correlation was also computed, prioritizing those genes that had the strongest negative correlation (anti-correlation). Anti-correlation between miRNA and mRNA expression profiles can likely mean there is a miRNA target interaction. Compared to other proposed approaches for the analysis of miRNA and target expression profiles, such as Pearson correlation and Lasso, the global test provided better results regarding sensitivity and specificity, as proved in [28]. The global test has been computed using the “globaltest” R library [29].

### Shiny package

Shiny [30] is an R package which makes possible build and deploy web server applications using R language. This package is delivered as a component that runs over the RStudio server development environment [31]. Shiny introduces a suite of the most common graphical components for interactive data visualisation, as well as the latest bootstrap responsive template. Moreover, it allows designing web applications that are built entirely using R. Since R is the development language for both the web-service and the data analysis, the use of Shiny introduces some advantages. Shiny, in fact, enables R to re-run its expressions in the back-end whenever the user makes a change in the front-end side (the web application user interface). Similarly, as regards data visualisation, Shiny allows interacting with the graphical components of the user interface, which in turn can modify linked R data, such as variables or data frames. The change of R data involves the re-running of some portions of R code and, consequently, an update of some specific graphs and/or tables in the front-end side.

### miRNA-target interaction types

All the miRNA target interactions we analysed, are classified in 4 possible interaction types: degradation, inhibition, no interaction and no interaction*. That assignment is given by exploiting the correlation analysis among miRNA-gene and gene-protein expression profiles, using the global test as described in the previous Section. The resulting schema is reported (Fig. 2). “Inhibition” result is given by a positive correlation between miRNA and gene and an anti-correlation between gene and protein. This second correlation analysis allows considering miRNA-gene couples that in other prediction algorithms, based only on miRNA-genes expression profiles, would be lost. No interaction types are if both miRNA, gene and protein are correlated. For those cases where there is no data available on protein expression, the interaction type is called “no interaction*”. “degradation” result is given by a negative correlation between miRNA and gene, and a positive correlation between gene and protein. Notice that the type of interaction result called “degradation” also includes cases showing miRNA-gene couples that are anti-correlated and gene-protein couples that are also anti-correlated. This choice, can be apparently in discordance with miRNA behaviour towards its target, but it can be justified by the evidence that mRNA abundance levels cannot be correctly used as proxies for corresponding proteins concentrations and activities, because of different dynamic processes as mRNA processing, localization, post-transcriptional modifications of the proteins themselves [32]. Indeed, different scientific papers have long debated about the correspondence between transcript levels of a given gene and its coded protein [33–37], and numerous reports have concluded that proteome and transcriptome abundances are not enough correlated to act as proxies for each other [32, 38].

## Results

In this section, we present some case studies solved through the use of miRTissue web application. In the first part of the Section, we describe in what we call “standalone scenarios” how miRNA, gene and protein expression values can help to better understand the molecular behaviour of miRNA-target interactions in different tissue contexts, just using miRTissue in a standalone way. In the second part of the Section, we present how of miRTissue can be adopted in common computational pipelines to improve the investigation of current bioinformatics tasks. We define those scenarios as “bioinformatics scenarios”.

In more detail, standalone scenarios focus on (1) miRNA-gene-protein interaction analysis in breast cancer tissue, (2) miRNA-gene-protein comparison analysis in two different tumor types, and (3) comparative study between normal and cancer tissues in two different tissue types. Bioinformatics scenarios deal with (1) miRNA therapeutics analysis cancer, (2) biomarker discovery in cancer, (3) miRNA-protein interaction for network analysis.

### Standalone scenario 1: miRNA-target-protein analysis in breast cancer

Considering the “miRNA-target-protein analysis for a specific tumor type” for the breast cancer tissue (BRCA), the miRTissue service will provide 3921 miRNA-target verified interactions where protein expression values are given. The most of interaction types are “degradation” of mRNA (1774 entries), whereas 50 entries report “inhibition” and the remaining results are “no interaction”.

**Fig. 3:**
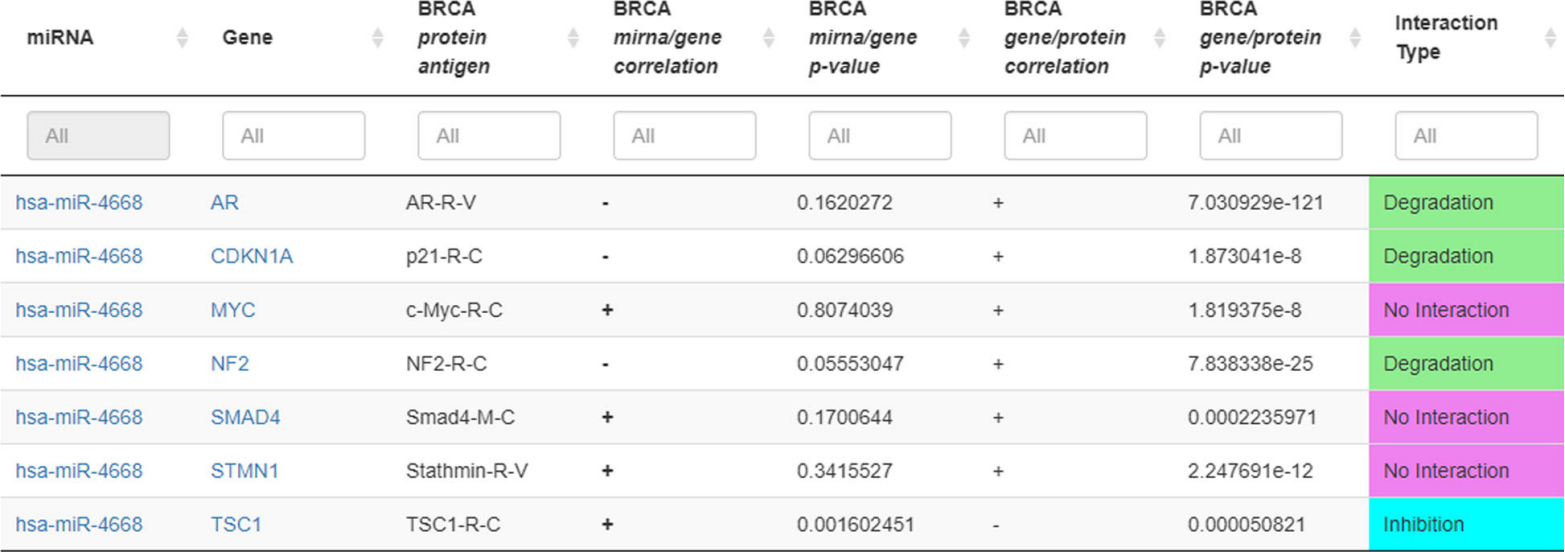
Standalone scenario 1: miRNA-target-protein analysis of BRCA tumor tissue. The screenshot shows all the BRCA tumor tissue miRNA-gene interactions for the hsa-miR-4668 miRNA, when only miRNA gene pairs where protein expression values are given is taken into account. It is possible to notice that considering both miRNA-gene and gene-protein correlations, the reported pairs belong to three different interaction types

A clear contribution introduced by miRTissue is the evidence of miRNA-target interactions that could be lost if we only consider miRNA and gene profiles without considering protein expression values. For instance (Fig. 3), miRNA hsa-miR-4668 interacts with TSC1 gene, causing inhibition of protein translation (miRNA/gene *p*-value: 1.60*e*−3; gene/protein *p*-value: 5.08*e*−5). This result is confirmed by scientific literature [39]. Indeed, Jiang et al. showed aberrant expression of TSC gene in breast cancer that it is related to clinical outcome in this cancer tissue [40]. Moreover, Karginov et al. showed a remodelling of Ago2-mRNA interactions upon cellular stress reflecting miRNA complementarity and correlating with altered translation rates [41]. The output of the discussed analysis allows to better understand the different behaviour that a miRNA can have on its target and to avoid missing potentially relevant information.

### Standalone scenario 2: miRNA-gene-protein comparison analysis in two different tumor types

**Fig. 4:**
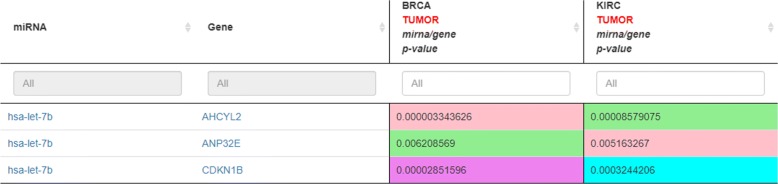
Standalone scenario 2: miRNA-target analysis of BRCA vs. KIRC tumor tissues. Here three miRNA-target interactions for two different tumor types are compared. This evidence how the tissue-specificity can change the behavior of the same miRNA-gene pair. The biological meaning of the color associated to *p*-value cells is reported in Fig. 2

Using the miRTissue service, the user can analyse and compare miRNA-target interactions for more than one cancer type. If we analyse both breast cancer (BRCA) and kidney renal clear cell carcinoma (KIRC), interestingly, the web application returns a different interaction type for the same miRNA-target pairs (Fig. 4). For instance, the output of the analysis for the couple hsa-let7b and CDKN1B is “no interaction” when the BRCA tissue is considered, and “inhibition” of the protein translation when the KIRC tissue is taken into account. The same for the other two miRNA-target pairs. Once again, this result evidences the importance of integrating validated interactions with expression values, which allow showing biological results not detected with other services.

### Standalone scenario 3: a comparative analysis between normal and cancer tissues

**Fig. 5:**
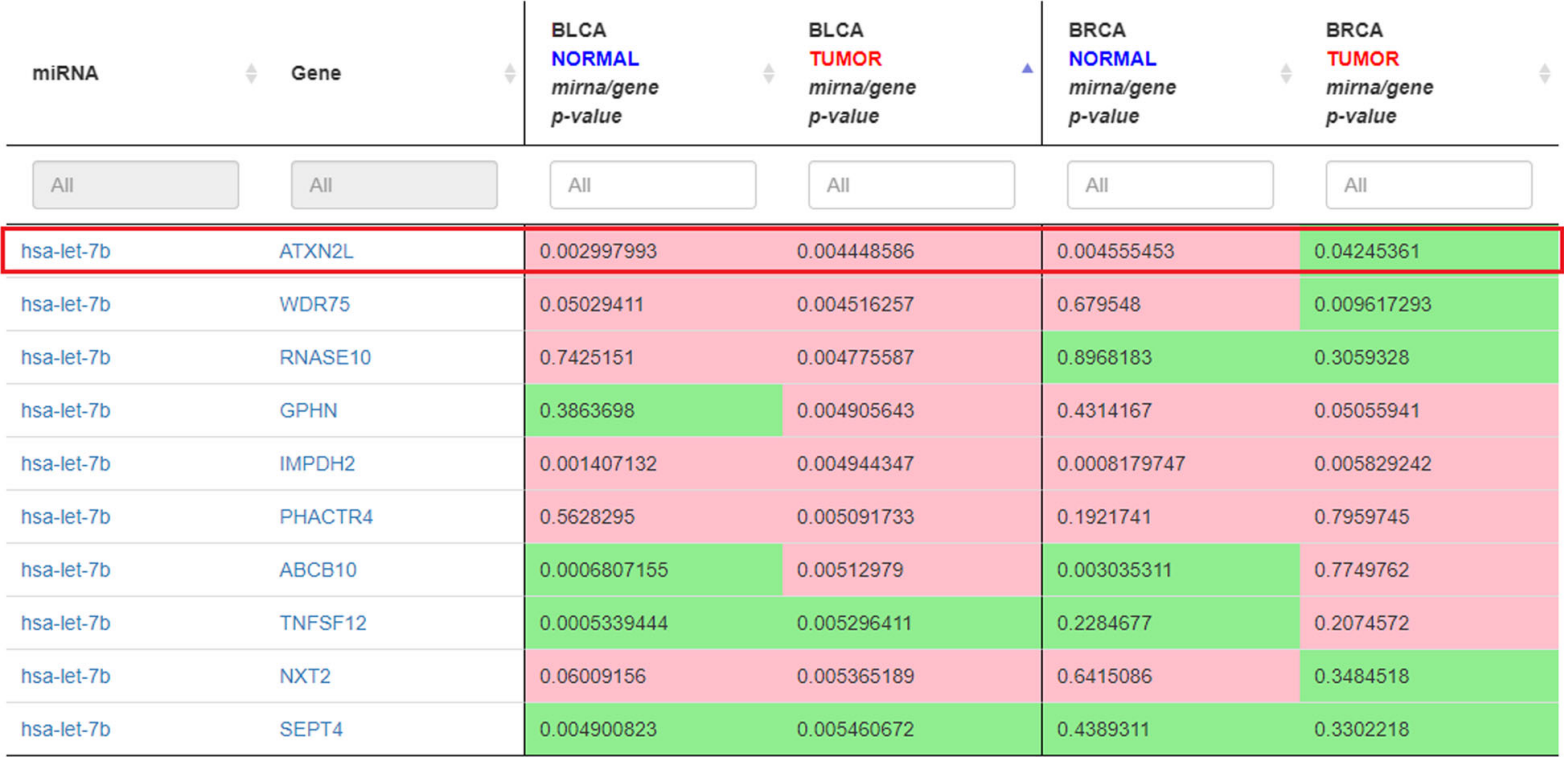
Standalone scenario 3: miRNA-target analysis of BLCA vs. BRCA normal/tumor tissues. Here we simultaneously compare different tissue types both of healthy and tumor conditions. For instance, the red box highlights a miRNA-target pair where the interaction type differs when two different conditions as the physiological (normal) and pathological (tumor) one are taken into account, also for two different tissue type. Although the highlighted interaction could not be relevant for the BLCA tissue, it can have a different significance when BRCA tissue is analyzed, since miRNA acts degradating its target in tumor type, compared to breast normal tissue

miRTissue can also allow doing comparative analysis between normal and cancer tissues for a specific tumor type, or to compare simultaneously different tissue types both of healthy and tumor conditions, using the “normal/tumor miRNA-target analysis” feature. Considering invasive breast carcinoma (BRCA), and bladder urothelial carcinoma (BLCA), the proposed service can easily detect different behaviour for the same miRNA-target couple (Fig. 5). For the specific case of hsa-let-7b and ATXN2L gene, there is no interaction both in normal tissue and bladder urothelial carcinoma (miRNA/gene *p*-value in bladder normal tissue: 3.0*e*−3, miRNA/gene *p*-value in bladder urothelial cancer: 4.45*e*−3), instead in breast carcinoma miRNA interacts with its target gene causing its degradation (miRNA/gene *p*-value in breast normal tissue: 4.55*e*−3, miRNA/gene *p*-value in breast cancer tissue: 4.24*e*−2).

### Bioinformatics scenario 1: miRNA Therapeutics analysis in cancer

**Fig. 6:**
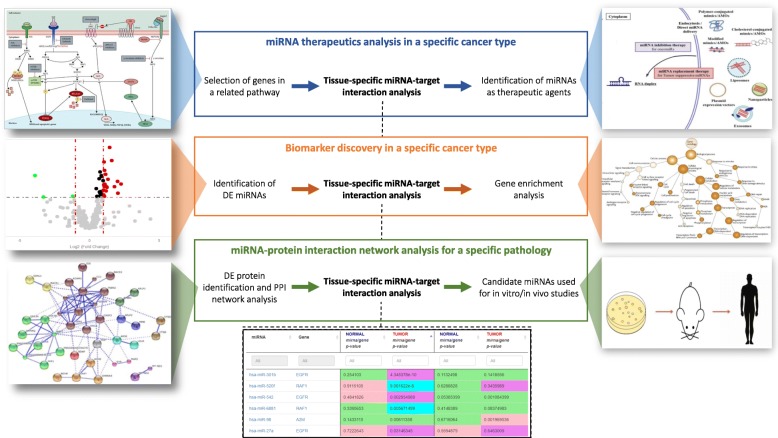
Bioinformatics scenarios: how miRTissue can help in different computational pipelines. We present how of miRTissue can be adopted in common computational pipelines in order to improve the investigation of current bioinformatics tasks. The upper part refers to “miRNA Therapeutics analysis in cancer”, the central part refers to “biomarker discovery in cancer”, the lower part refers to “miRNA-protein interaction for network analysis”

An emerging application of miRNAs in translational medicine is their use as therapeutic agents in tumor disease. Thanks to their dual role as oncogenes or tumor suppressors, it is possible to build synthetic miRNA molecules called miRNA-mimics or AntimiRs respectively, depending on the function of the target gene (respectively tumor-suppressor and oncogene) they interact with. The former mimics the function of corresponding miRNAs, the latter acts as miRNA-antagonists [42]. Moreover, another feature that makes these synthetic miRNAs interesting candidates as therapeutic agents is the ability of a single miRNA to target multiple mRNAs. That implies a single miRNA therapeutic agent can act on different targets at the same time. There are already several microRNAs-based therapeutic strategies in cancer, as these small RNA molecules could have the advantage to be injected easily through parenteral injection [43]. Considering a cancer type, we want to discover miRNA molecules used as potential therapeutic agents in that cancer tissue. The analysis requires different steps, described as follows: 1) selection of genes belonging to the cancer pathway, 2) miRNA-target interaction analysis, 3) miRNA analysis and validation as miRNA therapeutics through in vitro or in vivo experiments (Fig. 6, upper part). In this context, the use of miRTissue web service contributes to specifically select miRNAs that are experimentally validated to interact with previously selected targets in a specific tissue type. This feature allows having less false positive results, giving a more restricted list of miRNAs to test in the laboratory. Moreover, this implies a cost reduction and less time-consuming.

### Bioinformatics scenario 2: biomarker discovery in cancer

miRNAs are small RNA molecules with a big potential in clinical applications. They are dysregulated in several physiological and pathological conditions as cancer [3]. Different studies show their potential use as biomarkers both in diagnosis (cancer type classification and differential diagnostics of malignant nodules compared with benign forms), and therapy (selection of patients according to molecular features for targeted therapies; radio-resistance assessment and monitoring treatment response and early detection of relapse after therapy) [44]. Defining specific biomarkers strictly linked to specific tissues will allow refining and better focus on the therapeutic strategies of diagnosis and intervention in cancer disease. The proposed case study focuses on the identification of differentially expressed (DE) miRNAs in cancer tissue and gene enrichment analysis. To solve the proposed case study the following steps are required: 1) identification of DE miRNAs, 2) miRNA-target interaction analysis, 3) gene enrichment analysis of identified targets (Fig. 6 central part). Once computed the DE miRNA list, it can be the input list for miRTissue. Then, it can be used to select just those gene targets interacting with miRNAs both in tumor and normal tissue types. The additional information on the normal tissue can give the opportunity to analyse different targets selectively, and point out different putative biomarkers related to both normal and tumor tissue types, highlighting this way different behaviours of the interacting molecules according to the tissue types.

### Bioinformatics scenario 3: miRNA-protein interaction network analysis in cancer

Interactome analysis has become one of the main focus of current biological research. In that field, protein-protein interaction (PPI) networks can provide much information about the comprehension of biological processes in an organism, and their aberrant interaction networks are the basis of multiple aggregation-related diseases, such as Creutzfeldt–Jakob, Alzheimer’s diseases, and may also lead to cancer [45]. Moreover, a PPI network is not a static entity, but it is a dynamic process since it is functionally related to gene expression and consequently to regulatory mechanisms. In this context, miRNAs play a fundamental role, as they act as negative regulators of gene expression. For those reasons, there is a need to enlarge this network analysis to further regulative elements, to better understand all the potential connections between those elements, and to better define the molecular aetiology of diseases, as well as the discovery of putative biomarkers in different diseases. The analysis requires different steps, described as follows: 1) identification of DE proteins, 2) PPI network creation, 3) miRNA-target interaction analysis, 4) gene ontology analysis, 5) pathway analysis. Supposing to identify a list of DE proteins, a PPI network can be produced. miRTissue allows to do target selection and identification of interacting miRNAs, applying *p*-value thresholds as well as filtering for tissue specificity. Then the identified list of miRNAs can be used to do gene ontology enrichment and pathway analysis aiming to draw attention to miRNA-protein network connections, and potential tumour-suppressor/oncogenic miRNA functions linked to specific cellular pathways (Fig. 6, lower part).

Indeed, miRTissue can highlight miRNA behaviour, as miRNAs causing translation inhibition can be shown. These in silico steps would simplify the choice of miRNA candidates to be applied for vitro or in vivo experiments, as for example in the study of potential biomarkers for specific tumor diseases.

## Discussion

One of the main obstacles in miRNA research is to have a high grade of specificity and sensitivity in miRNA-target interaction analysis [19]. Recently, some bioinformatics tools, either miRNA-target database and predictors, began to integrate expression values of interacting molecules [46] because of the observation that RNA molecules (both miRNAs and mRNAs) are differentially expressed in different tissues. This implies that if a particular miRNA-target couple is validated and/or predicted in a specific tissue type, the same interaction could not be true either for a different tissue. To overcome this issue, we propose the miRTissue web application that joins expression profiles of miRNAs, genes, and proteins, with validated interactions. Although miRTarBase, as a database of validated miRNA-target interaction, and some predictors, such as miRGator, gives info about expression profiles in different tumor tissues to characterize the interactions better, miRTissue offers several improvements. First of all, protein expression data allows to also take into account interactions causing translation inhibition eventually. Indeed, differently from all the other miRNA-target predictors, miRTissue offers information about the interaction type, that is mRNA degradation or translation inhibition. Moreover, with respect to the above-cited tools, miRTissue allows to directly make queries filtering the interactions for tissue type, among 15 kinds of cancer, and tissue condition, i.e. tumor and normal. Finally, the use of the global test for computing correlation among expression profiles gives better results regarding sensitivity and specificity of the considered interactions, as stated in [28], with regards to the Pearson correlation implemented in miRTarBase and miRGator. The proposed web application offers some improvement for complex analysis, providing information about interaction type and tissue specificity, both in a standalone use, that we called standalone scenarios, as well as in bioinformatics computational pipelines, that we called bioinformatics scenarios.

From a numeric perspective, starting from about 340k uncharacterised validated miRNA-target interactions extracted from miRTarBase, an average of just 160k validated miRNA-target interactions per cancer type belong to “Degradation” or “Inhibition” interaction types. That means the use of miRTissue allows having the advantage to leave out about 54% of the initial interactions set for the analysis of a specific tissue.

## Conclusion

In this work, we presented miRTissue, a publicly available web application that introduces some advantages with respect to the state-of-the-art services dealing with miRNA-target interactions. First of all, considering both validated targets and the expression values of different RNA molecules in different tissues, it allows having less false positives, providing a deeper view of a tissue’s RNA status. Moreover, to the best of our knowledge, miRTissue is the first service that integrates protein expression values, giving additional information on miRNAs behaviour concerning the other tissue-specific miRNA-target services. The miRTissue web application also allows the user to easily explore and compare a different kind of tissues or couples of miRNAs and genes, through an intuitive and handy user interface. Finally, miRTissue contains only validated interactions instead of predicted ones, allowing to strengthen the results of our pipeline for further analysis.

As future work, we plan to integrate predicted miRNA-target interactions from different online databases to increase miRNA gene pairs to be characterised with our method, as well as to add another type of tissues (when available).

## Additional file

Additional file 1 Technical and implementation details of the miRTissue software architecture. (PDF 819 kb)

## Abbreviations

BLCA: Bladder urothelial carcinoma
BRCA: Breast cancer
CDS: Coding sequence
DB: Database
DE: Differentially expressed
KIRC: Kidney renal clear cell carcinoma
miRNA: microRNA
mRNA: RNA messenger
NCI: National cancer institue
NHGRI: National human genome research institute
PPI: Protein-protein interaction
TCGA: The cancer genome atlas
UTR: Untraslated region
