# Supplementary material for: miRTissue: a web application for the analysis of miRNA-target interactions in human tissues
Source: BMC Bioinformatics. 2018 Nov 30;19(Suppl 15):434. doi: 10.1186/s12859-018-2418-5 (PMC6266954; doi:10.1186/s12859-018-2418-5)
Supplement: Supplementary file 1 — Technical and implementation details of the miRTissue software architecture. (PDF 819 kb) [file 12859_2018_2418_MOESM1_ESM.pdf]

Supplementary material S1: technical and implementation details

1 Computational pipeline

To provide the interaction types related to specific tissues for each couple of validated miRNA-target interaction, miRTissue implements the following pipeline (Figure 1). For each pair of miRNA-target interaction in human extracted from miRTarBase, corresponding expression profiles of miRNA, mRNA and related proteins are collected from TCGA platform using the TCGAbiolinks R library [1]. Only those miRNA-target pairs with both molecules profiled in TCGA datasets are kept in our system. Then the global test for correlation estimation is computed following the same approach presented in [2]. Given a miRNA, we computed the global test among miRNA expression profile (representing the response variable) and the expression profiles of all of its target genes (representing the covariates). This procedure is repeated for each tumor and normal tissue. As a result, we obtain for each miRNA-gene pair a correlation sign and the corresponding p-value, that is the statistical significance. Global test is then also computed between mRNA expression profiles and its corresponding protein expression profile in order to estimate gene-protein expression correlation.

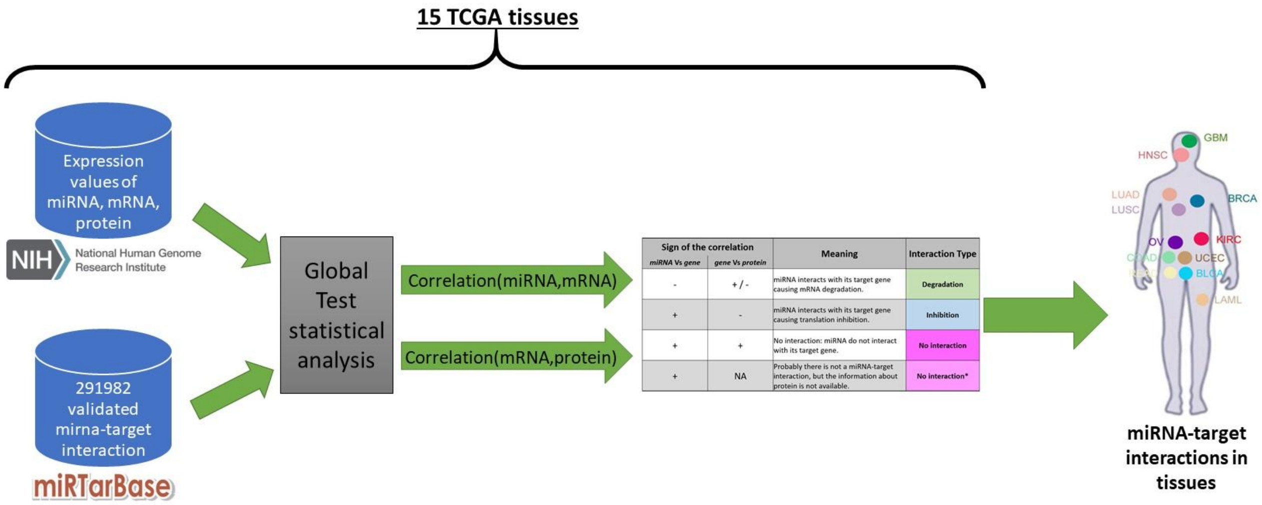

Figure 1: Starting from a set of validated miRNA-target interactions and a collection of miRNA, gene and protein expression values from different tissue samples, the proposed pipeline provides the interaction type using the global test statistical analysis.

2 Web application

In this section, we introduce the miRTissue web application, which allows users to explore data obtained in the previous steps from different points of view. Here, we report the architecture we adopted to develop and deploy the web application, and then we describe the user interface. miRTissue is available at the following URL: http://tblab.pa.icar.cnr.it/mirtissue.html

2.1 Architecture

The miRTissue web application was implemented following a typical three-tier architecture [3], i.e. it is composed of three layers: user interface (UI) layer, business logic (BL) layer and data access (DA) layer. BL layer handles the computation of the global test and the storing of the results on the database, as well as the management of the services' control flow. BL is implemented through a set of R scripts.

Similarly, to implement the UI layer, we used the R Shiny package, that contains a large set of layouts and graphical components that are able to generate a UI object that is converted by the Shiny server in a web

document. Finally, as regards DA layer, we stored data in a MySQL database management system [4] and we adopted the RODBC library [5], implementing the ODBC database connectivity, to perform the database query. An implementation view of the proposed web application is shown in Figure 2. Here the main code runs on the Shiny server, that is connected to some external resources, such as static HTML pages, the MySQL database mentioned above and some utilities (R packages). Users can interact with the miRTissue application through every web browser.

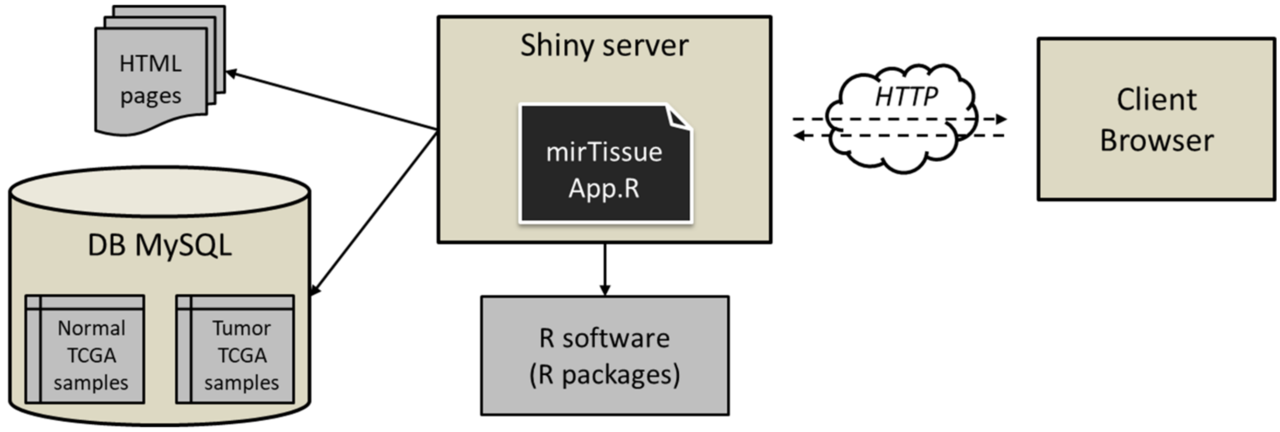

Figure 2: An implementation schema of the proposed service

2.2 Web user interface

Here we discuss the main characteristics and features of miRTissue web user interface. In detail, the user interface is composed of three tabs: two of them allow users to select, explore and download all the information about validated miRNA-target interactions in different tissues, whereas the last one shows external resources details. We introduce further information in the following subsections.

2.2.1 Normal/Tumor miRNA-target analysis

This panel is composed of a sidebar that contains four query filters, and the main panel, that contains the miRNA-gene interaction(s) output table. In detail, the sidebar allows the user to select: (1) a set of miRNAs, (2) a set of target genes, (3) a set (at least one) of TCGA tissue and (4) the tissue type. To help the user to write the right entries, we add an auto-completion drop-down list for both miRNAs and targets; if both lists are left empty, all the miRNA-target interactions will be taken into account. Finally, the fourth input field, the tissue type radio buttons, allows to select normal and/or tumor tissues; in case both of them are selected, the output table can be used as a comparison table that shows interactions with different behaviour in the tumor condition concerning the normal one. As regards the main panel, it shows the result of the user query as a data table, containing for each selected TCGA tissue, the p-value of the correlation between each miRNA-target pair. Also, each cell of the data table is coloured accordingly, to provide information about interaction type, i.e. degradation, inhibition or ``no interaction''. Results can be ordered and filtered for specific p-value ranges and exported as a comma-separated values (CSV) file or as a Microsoft Excel sheet for further analysis. Looking at a sample caption of this panel (Figure 3), it is possible to note the correlation among miRNA-target interactions in three different TCGA tissues (BRCA, LUAD and PRAD), for both normal and tumor tissue types.

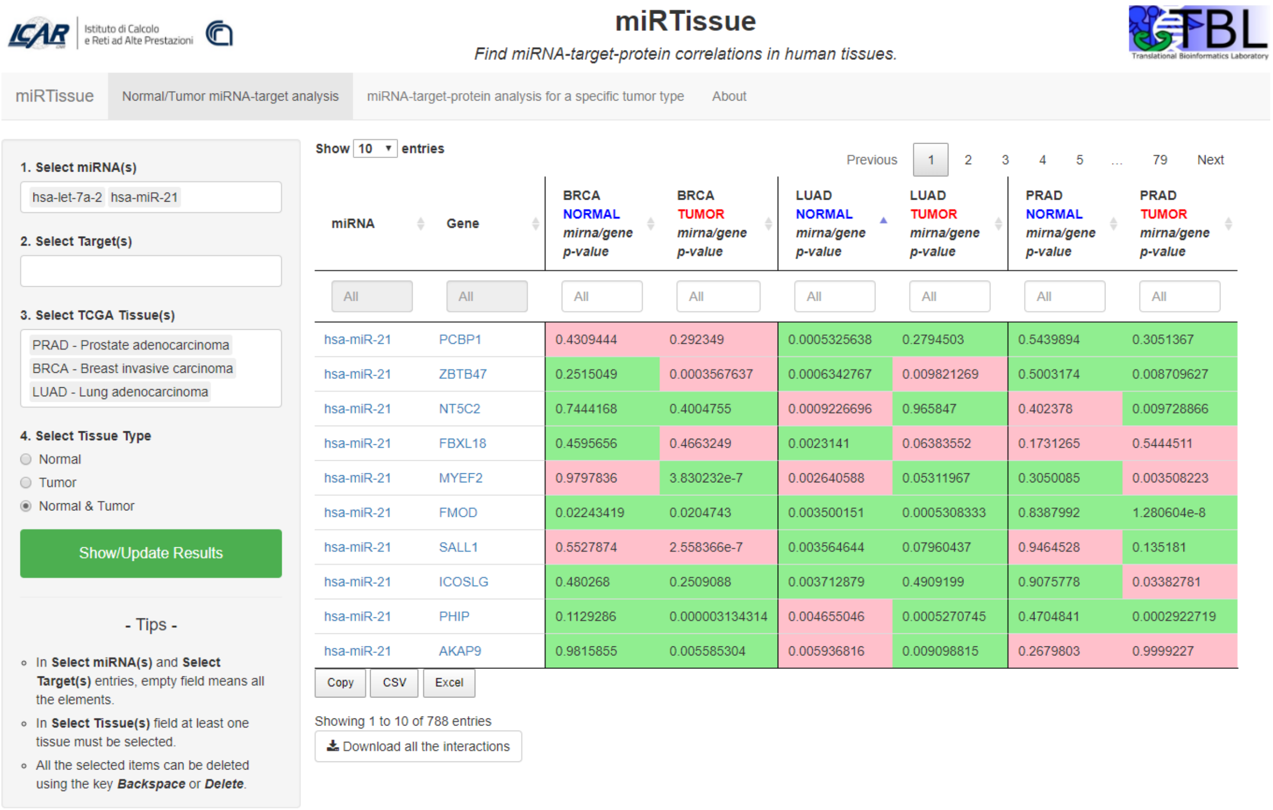

Figure 3: A screenshot of the ``Normal/Tumor miRNA-target analysis'' feature of the proposed web application.

2.2.2 miRNA-target-protein analysis for a specific tumor type

This panel allows exploring in detail all the miRNA-target interactions of a single tissue, providing an immediate visualisation of the interaction types. It is composed of a sidebar, containing four query filters, and the main panel, showing the miRNA-gene interaction(s) output table. As well as the previous panel, also this one requires four inputs, i.e. (1) a set of miRNAs, (2) a set of target genes, (3) a specific TCGA tissue and (4) a filter that allows showing only interactions where protein expression value is given. Once again, both miRNAs and targets filters are auto-completion drop-down lists. A sample caption of ``Tumor tissue miRNA-gene-protein analysis'' tab is reported (Figure 4), showing all the interactions involving two miRNAs (miR-hsa-21 and miR-let-7a) in the adrenocortical carcinoma (ACC) tissue. There, the main panel shows detailed information about each interaction, including the antigen related to the protein, the correlation signs of both miRNA Vs gene and gene Vs protein and the p-value associated to these correlations. Finally, the last column of the output table provides the interaction types (i.e., degradation, inhibition or ``no interaction''). Concerning the previous panel, here it is possible to order and filter all the results also for the correlation sign or interaction type.

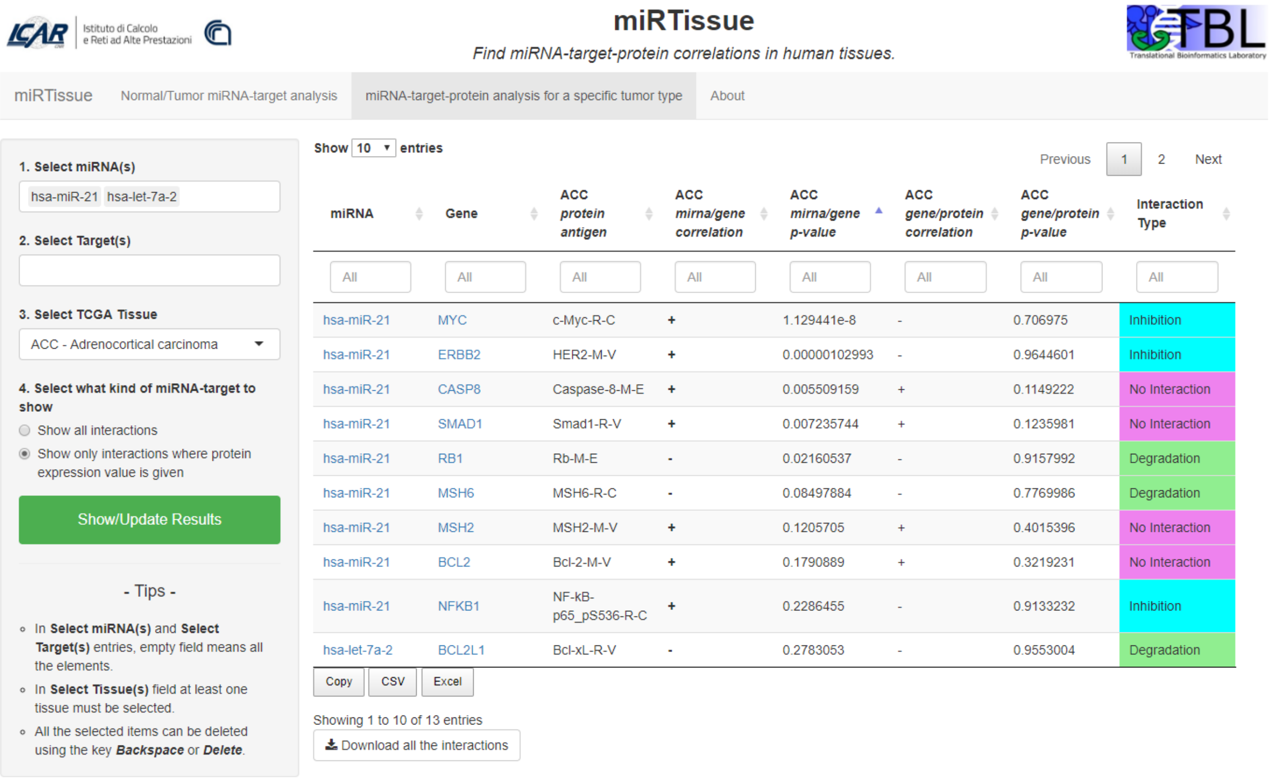

Figure 4: A screenshot of the ``miRNA-target-protein analysis for a specific tumor type'' feature of the proposed web application.

2.2.3 About

This panel contains a list of the external resources, algorithms, libraries and tools we used to implement miRTissue web application. For each resource, we also included the release we adopted. Finally, we added a reference to how to contact us.

References

[1] A. Colaprico, T. C. Silva, C. Olsen, L. Garofano, C. Cava, D. Garolini, T. Sabedot, T. M. Malta, S. M. Pagnotta, I. Castiglioni, M. Ceccarelli, G. Bontempi, and H. Noushmehr, "TCGAbiolinks: An R/Bioconductor package for integrative analysis of TCGA data," *Nucleic Acids Res.*, 2015.

[2] M. van Iterson, S. Bervoets, E. J. de Meijer, H. P. Buermans, P. A. C. 't Hoen, R. X. Menezes, and J. M. Boer, "Integrated analysis of microRNA and mRNA expression: adding biological significance to microRNA target predictions," *Nucleic Acids Res.*, vol. 41, no. 15, pp. e146–e146, Aug. 2013.

[3] C. Caragea, V. Honavar, P. Boncz, P. Boncz, P.-Å. Larson, et al., "Multi-Tier Architecture," in *Encyclopedia of Database Systems*, Boston, MA: Springer US, 2009, pp. 1862–1865.

[4] M. Widenius and D. Axmark, *Mysql Reference Manual*, 1st ed. Sebastopol, CA, USA: O'Reilly & Associates, Inc., 2002.

[5] B. Ripley and M. Lapsley, "RODBC: ODBC Database Access." 2017.
